# Protocol for stage 1 of the GaP study (Genetic testing acceptability for Paget's disease of bone): an interview study about genetic testing and preventive treatment: would relatives of people with Paget's disease want testing and treatment if they were available?

**DOI:** 10.1186/1472-6963-6-71

**Published:** 2006-06-08

**Authors:** Anne L Langston, Marie Johnston, Clare Robertson, Marion K Campbell, Vikki A Entwistle, Theresa M Marteau, Marilyn McCallum, Stuart H Ralston

**Affiliations:** 1 Health Services Research Unit, University of Aberdeen, Polwarth Building, Foresterhill, Aberdeen, AB25 2ZD, UK; 2 Dept of Health Psychology, University of Aberdeen, Polwarth Building, Foresterhill, Aberdeen, AB25 2ZD, UK; 3 Social Dimensions of Health Institute, University of Dundee, 11 Airlie Place Dundee, DD1 4HJ, UK; 4 Psychology Department (at Guy's), Health Psychology Section, Psychology and Genetics Research Group, 5th Floor Thomas Guy House, Guy's Campus, London Bridge SE1 9RT, UK; 5 National Association for the Relief of Paget's Disease, 323 Manchester Road, Walkden, Worsley, Manchester, M28 3HH, UK; 6 Molecular Medicine Centre, University of Edinburgh, Western General Hospital, Edinburgh, EH4 2XU, UK

## Abstract

**Background:**

Paget's disease of bone (PDB) is characterised by focal increases in bone turnover, affecting one or more bones throughout the skeleton. This disrupts normal bone architecture and causes pain, deformity, deafness, osteoarthritis, and fractures.

Genetic factors are recognised to play a role in PDB and it is now possible to carry out genetic tests for research. In view of this, it is timely to investigate the clinical potential for a programme of genetic testing and preventative treatment for people who have a family history of PDB, to prevent or delay the development of PDB.

Evidence from non-genetic conditions, that have effective treatments, demonstrates that patients' beliefs may affect the acceptability and uptake of treatment. Two groups of beliefs (illness and treatment representations) are likely to be influential.

Illness representations describe how people see their illness, as outlined in Leventhal's Self-Regulation Model. Treatment representations describe how people perceive potential treatment for their disease. People offered a programme of genetic testing and treatment will develop their own treatment representations based on what is offered, but the beliefs rather than the objective programme of treatment are likely to determine their willingness to participate. The Theory of Planned Behaviour is a theoretical model that predicts behaviours from people's beliefs about the consequences, social pressures and perceived control over the behaviour, including uptake of treatment.

**Methods/design:**

This study aims to examine the acceptability of genetic testing, followed by preventative treatment, to relatives of people with PDB. We aim to interview people with Paget's disease, and their families, from the UK. Our research questions are:

1. What do ***individuals with Paget's disease ***think would influence the involvement of their relatives in a programme of genetic testing and preventative treatment?

2. What do ***relatives of Paget's disease sufferers ***think would influence them in accepting an offer of a programme of genetic testing and preventative treatment?

**Discussion:**

Our research will be informed by relevant psychological theory: primarily the Self-Regulation Model and the Theory of Planned Behaviour. The results of these interviews will inform the development of a separate questionnaire-based study to explore these research questions in greater detail.

## Background

### The need for this research

Over recent years, advances in human molecular genetics have resulted in the identification of polymorphisms and mutations in several genes that cause or predispose to diseases such as cancer, neurodegenerative conditions and inborn errors of metabolism. For some of these conditions, such as Huntington's disease and muscular dystrophy, it is possible to offer a genetic test that will give information on the probability of the disease occurring but this is somewhat unattractive to patients without the prospect of an effective treatment. For other diseases, such as hypercholesterolaemia and haemochromatosis, identification of genetic susceptibility can be used as a risk factor to inform management strategies and treatment decisions in a similar way to other clinical risk factors.

This application aims to test the feasibility of translating genetic knowledge into practice by examining the acceptability of offering genetic testing, followed by therapeutic intervention, to people with Paget's disease of bone.

### Paget's disease of bone

Paget's disease of bone (PDB) affects about 3% of individuals over the age of 55 years in the UK [1]. It is characterised by focal increases in bone turnover, affecting one or more bones throughout the skeleton. The abnormal bone turnover disrupts normal bone architecture and causes bone pain, bone deformity, deafness, osteoarthritis, and pathological fractures [2]. Genetic factors have long been recognised to play an important role in Paget's disease [3-6] and recent studies by our own group and others indicate that between 40–50% of instances of familial Paget's disease are caused by mutations affecting the ubiquitin-associated (UBA) domain of the *SQSTM1 *gene [7-11]. Genotype-phenotype analysis has shown that *SQSTM1 *mutations are highly penetrant, such that between 90–100% of individuals within families who carry mutations will have developed the disease by the age of 65 years [7-11].

In view of this, it is timely to investigate the clinical potential for a programme of genetic testing and preventative treatment for patients who carry *SQSTM1 *mutations, in an effort to prevent or delay the development of Paget's disease or its complications.

### Prospects for preventing Paget's disease

Bisphosphonates such as Risedronate, Pamidronate and Zoledronate have emerged as highly effective agents for reducing bone turnover and treating bone pain in patients with Paget's disease [12]. Clinical studies have shown that these drugs can restore elevated levels of bone turnover to normal in a high proportion of cases, can improve the appearance of Pagetic lesions on isotope bone scans [13], and can restore bone architecture to normal, as assessed histologically [14].

This raises the possibility that patients who carry *SQSTM1 *mutations and who are at high risk of developing Paget's disease could be offered prophylactic therapy, in an attempt to prevent the disease occurring, or to prevent complications developing. A particularly promising mode of treatment in this respect is Zoledronate, which is a highly effective treatment for Paget's disease even in microgram doses [15], with inhibitory effects on bone turnover that can extend for at least 12 months after a single injection [16]. There is also evidence to suggest that treatment with intravenous Pamidronate and oral Risedronate can suppress bone resorption in active Paget's disease for up to two years [17,18].

With the existence of effective treatment for Paget's disease that could prevent or delay the onset of the disease, we need to explore the acceptability of genetic testing followed by preventative treatment in patients with Paget's disease of bone.

### Possible factors that could affect acceptability of genetic testing and preventative treatment

Evidence from other areas of genetic testing suggests that patients may not wish to take genetic tests. For example, in Huntington's disease, prior to the development of a genetic test, individuals at risk declared a much higher intention of taking a test than the actual numbers taking the test when it became available [19]. Studies have shown that there is a widespread belief that genetic conditions are not treatable. Such beliefs might affect the acceptability of diagnosis and treatment of genetic conditions. However, the additional offer of effective preventative treatment can make genetic testing more acceptable [20].

### Beliefs

Evidence from other, non-genetic, conditions, for which there are effective treatments, demonstrates that patients' beliefs may affect the acceptability and uptake of treatment. Two groups of beliefs (illness representations and treatment representations) are most likely to be influential.

#### Illness representations

Illness representations describe how people see their illness. This is most clearly outlined in the Self-regulation Model (SRM) [21]. The five key representations are:

• *identity *(e.g. the 'label', identifying symptoms);

• *cause *(e.g. stress, genetics);

• *consequences *(e.g. activity limitations, loss of wages);

• *timeline *(e.g. acute, fluctuating, progressing);

• *cure/control *(e.g. medication, diet, exercises).

In addition, the SRM identifies the *emotional representation *(e.g. frustrating, frightening) as a separate dimension of representation. Based on this model, Weinman and colleagues [22] have developed a measure, the Illness Perceptions Questionnaire (IPQ), to assess these representations and have found that they are predictive of uptake and response to a wide variety of treatments, including the bone disease Rheumatoid Arthritis [23]. Clearly, such beliefs may influence uptake of a new treatment offered to families with Paget's disease, and Marteau has shown that beliefs affect the decision to accept a genetic test [24].

Paget's disease has a highly variable presentation: age at onset, severity of symptoms and impact on sufferer's lives; and individuals might vary in their response to an offer of testing and treatment depending on the disease history within their family. Using the World Health Organisation International Classification of Functioning, Disability and Health model of health components [25], one might expect that all three components (impairment, activity limitations, and participation restrictions) might affect beliefs about the identity, consequences and timeline of the condition.

#### Treatment representations

**Figure 1:**
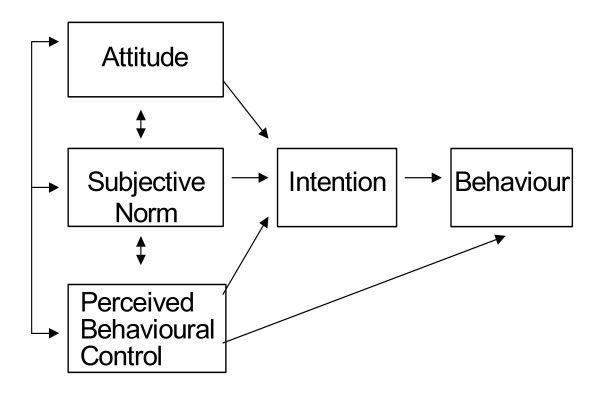
The Theory of Planned Behaviour (Azjen, 1991).

Treatment representations describe how people perceive potential treatments for their disease. There is considerable evidence that patients' beliefs about treatment (e.g. accessibility, burden, perceived likelihood of success) influence uptake and adherence [26]. People offered a programme of genetic testing and treatment will develop their own treatment representations based on what is offered, but the beliefs rather than the objective programme of treatment, are likely to determine their willingness to participate. The Theory Of Planned Behaviour (TPB) [26] (is a theoretical model, which predicts behaviours from people's beliefs about the consequences, social pressures and perceived control over the behaviour, including uptake of treatment (Figure 1).

### The nature of the treatment and treatment offer

The nature of the treatment, and the way in which the treatment is offered, may affect acceptability. One needs to consider not only how to invite the affected individual to 'involve' their family, but also how to introduce the programme to invited family members. Hardeman and colleagues [27] in the *Pro-Active *Programme, have developed an interview for families of people with Type 2 diabetes that incorporates the offer of the intervention for offspring. This interview was based on the interviews used to develop questionnaires for the Theory of Planned Behaviour (TPB) addressing beliefs about involving the offspring in the programme. It has proved very effective in rendering the programme acceptable [27].

In addition, the treatment offered for Paget's disease could be in tablet or infusion form, and could be administered in a range of environments. All of these factors may influence the acceptability of a programme of genetic testing and preventative treatment.

### Demographic and health characteristics

Demographic and health characteristics may also influence the acceptability of treatment. For example, someone who is older may think they are less at risk if they have passed the age of onset of Paget's disease in the affected person; or they may think they are nearing the time when the disease might affect them. Closeness to the affected person may make them view the condition as more or less serious, and they may be influenced by how the affected person copes with the illness. Furthermore, one might expect there to be more agreement within members of one family than between families due to their shared exposure to the condition as well as their opportunity to discuss and influence each other.

### Scientific value of this study

This project takes advantage of recent advances in knowledge about the molecular-genetic basis of PDB, with advances in therapeutics to address an important clinical question that is of relevance to patients with Paget's disease and their families. The project provides added value in that modern techniques in behavioural science will be applied to a specific issue in the treatment of Paget's disease, while at the same time investigating more general theories of human behaviour. Using the Self-Regulation Model, we will investigate how genetic information and preventive opportunities affect self-regulation via illness representations and, using the Theory of Planned Behaviour, how uptake of the offer of genetic testing and treatment is predicted by the constructs found to predict behaviour in other settings. Thus the findings will be relevant to these theories. They will also contribute to ongoing work to integrate these two models in understanding behaviour related to illness.

In addition, the knowledge gained from this investigation would be relevant to other musculoskeletal diseases such as osteoporosis, which also have a strong genetic component, and which can be prevented by bisphosphonates and hormone replacement therapy. With completion of the human genome project, and advances in human molecular genetics, it is probable that DNA testing with single genetic markers or combinations of markers will be offered for a wide range of other chronic diseases for which effective treatments are available. The results of the present research project will also be of direct relevance to the development of programmes of genetic testing and preventative intervention for these conditions.

### Study aim

The aims of the GaP study are to understand factors that might influence the acceptability of an offer of a programme of genetic screening and preventative treatment to families of Paget's disease sufferers.

The specific research questions are:

**1. **a) What do individuals with Paget's disease think would influence the involvement of their relatives in a programme of genetic testing and preventative treatment? and;

b) What do relatives of Paget's disease sufferers think would influence them in accepting an offer of a programme of genetic testing and preventative treatment?

**2. **Do the following factors affect acceptability of a programme of genetic testing and preventative treatment in relatives of Paget's disease sufferers?

• Illness and emotional representations of Paget's disease;

• Treatment representations;

• Presentation of the disease in affected relative(s) (age at onset, impairment, activity limitations, participation restrictions; family history as a function of the number of affected relatives and the relationship to the Subject);

• Respondent characteristics (age, gender and health);

• Beliefs of other family members; and

• The nature of the treatment offered.

By answering these research questions, this study will cast light on the feasibility of developing a programme of genetic testing and preventative treatment for individuals who carry *SQSTM1 *mutations that are at high risk of developing Paget's disease. Since we do not yet know whether prophylactic bisphosphonate therapy would be effective in preventing Paget's disease or its complications, we envisage that the results of the present study would be used to inform the design of a randomised controlled trial to investigate the efficacy of prophylactic bisphosphonate therapy in people with *SQSTM1 *mutations. Such a study would form the topic of a future research project.

### An overview of the GaP study

The GaP study has two stages:

Stage 1: This part of the study will address the first research question and aims to identify factors that would influence involvement of relatives not known to have Paget's disease in a programme of genetic screening and preventative treatment.

Stage 2: This part of the study is not part of the current protocol, and will comprise a postal questionnaire study of people without Paget's disease but who are relatives of people with Paget's disease.

## Methods/design for stage 1

Stage 1 will employ semi-structured interviews with individuals suffering from Paget's disease, and their relatives who have not been diagnosed as suffering from Paget's disease. The results of Stage 1 will inform the development of a TPB-based questionnaire for Stage 2.

The interviews with individuals suffering from Paget's disease will use the Theory of Planned Behaviour (TPB)-based [26].

### Subjects and recruitment

Individuals with Paget's disease will be identified from an established cohort: The PRISM trial.

The PRISM cohort involves 1331 patients with Paget's disease. The PRISM trial involves 39 collaborating centres ranging in size from 3 trial participants to approx 250 trial participants.

For this part of the study we will identify potential participants from 7 centres collaborating in the PRISM trial.

### Identifying subjects

A purposive sample of individuals with Paget's disease identified from the PRISM study will be invited to participate. Participating individuals will be asked to identify first and second-degree relatives without Paget's disease who they think might also be interested in taking part in the study. These individuals will be invited to participate in separate interviews. Relatives identified by this procedure will be selected for the study to represent a range of family relationships (e.g. siblings, children, grandchildren) and living conditions (e.g. living in same house, nearby, or far away). A sequential approach to sampling will therefore be used.

For this stage of the study, individuals will be approached from a restricted selection of PRISM centres (Table 1.).

These 3 groups of centres represent regions of the UK with varying incidences of Paget's disease. A low incidence of Paget's disease occurs in Scotland, whereas Manchester and Liverpool are well recognised as areas of high incidence of Paget's disease. Lancashire is considered to be the global 'hotspot' for Paget's. The southern England region represents an area with an incidence rate between those of the northern England and Scottish regions.

### Contacting subjects

A letter will be sent to probands inviting them to participate in an interview together with study information leaflets. The letter will include a 'tear off' section to return (in a reply paid envelope) if they are interested in discussing the study further. Probands will also be asked to provide contact details of one or two relatives who may also like to participate in an interview.

A letter will be sent to relatives identified by probands inviting them to participate in an interview together with study information leaflets. The letter will include a 'tear off' section to return (in a reply paid envelope) if they are interested in discussing the study further.

A researcher will telephone those who return the slip, and who are interested in taking part, to discuss the study further. If the person is still interested, an appropriate time and place for interview, and arrangements for formal consent will be discussed. There will be no follow-up of those probands or relatives that decline to participate or who do not reply.

A Thank You letter will be sent to probands and relatives taking part in Stage 1, once each individual interview is completed.

### Sample size

Sampling will continue until saturation, i.e. no 'new' ideas are being introduced within the three TPB beliefs (behavioural, normative and control) and 5 SRM domains (identity, cause, consequences, cure/control, timeline) plus emotional representations). It is expected that this will involve 10–20 individuals from the PRISM cohort together with 10–20 relatives of these affected individuals.

Purposive sampling will be used to identify probands and relatives to be approached for participation in this stage. Subjects are not chosen at random but instead are chosen because they are expected to facilitate investigation of the range of views and opinions relevant to the research. The aim of sampling is therefore to maximise variety for analysis. Subjects will be purposively selected to include a good spread across the following factors:

### Probands

• Gender (male, female);

• Age (<40 years, 41–60 years, >61 years);

• Severity of disease (monostotic, polyostotic);

• Time elapsed since diagnosis (<5 years, > 5 years); and

• Family history (Yes, No).

### Relatives

• Gender (male, female);

• Age (<40 years, 41–60 years, >61 years);

• Severity of disease in proband (monostotic, polyostotic);

• Relationship to proband (parent, sibling, grandparent, aunt/uncle); and

• Living proximity to proband (within 50 miles, further than 50 miles).

Anne Langston will undertake selection of interviewees, and the interviewer (Clare Robertson) will be blinded to these details.

### Exclusion criteria

Exclusion criteria include:

• Known* limited life expectancy (under one year); or

• Aged less than 18 years.

* *The caveat of 'known' is used, as it may not be possible to determine limited life expectancy of relatives*.

A lower age limit has been imposed (minimum age of 18 years). Clinical studies suggest that people with *SQSTM1 *mutations develop Paget's disease from approximately 45 years of age onwards [9]. Clinical intuition would suggest that starting a programme of preventative treatment at age 40 years would be therefore be appropriate. However, the clinical evidence to support this is still lacking. In addition, it is likely that a psychologically 'acceptable' age for beginning preventative treatment will differ from that indicated by existing evidence. As such, we propose to include all adult relatives of probands to determine the effect of age of the acceptability of genetic testing, and preventative treatment.

### Special arrangements for including people with specific communication needs

We have not made special arrangements for non-English speaking subjects for two reasons:

1. Providing multi-lingual facilities for this small number of interviews will be logistically and financially prohibitive;

2. Paget's disease is rare in non-Caucasians and therefore we anticipate that English will be the first language of the majority of eligible individuals within the UK.

3. It has not proved necessary to provide special arrangements for participants of the PRISM trial, and therefore it is not anticipated that the requirement will exist for this study.

Participants whose first language is not English will not automatically be excluded from the study if they are either able to communicate in English, or an interpreter is available to assist with communication. Such arrangements will be accommodated, if it is practicable to do so, on an individual basis.

Individuals with limited life expectancy are excluded from Stage 1 of the study as the interview process may be cognitively and emotionally taxing for them.

Individuals with known limited hearing ability may be excluded from Stage 1 due to practical difficulties of carrying out an interview and the distress and frustration this may cause the participant. If a participant is deaf but can lip-read, or has a friend or family member who can use Sign Language for them, the individual will not be excluded. Since deafness caused by Paget's disease of the skull is an important complication of Paget's disease, every attempt will be made to include these individuals in Stage 1 of the study, where practicable and where it will not cause distress to the participant.

Information leaflets and consent forms will be made available in large print upon request.

### Setting

For Stage 1, identified PRISM probands and relatives will be invited to interview at home or in a convenient clinical or research environment. The place of the interview will be according to the subject's preference. Travel expenses incurred by the subject will be reimbursed. Interviews will be tape-recorded and will be conducted face-to-face to allow the interviewer to counsel for consent.

### Consent process

Consent will be sought at interview and participating probands and relatives will be asked to sign a consent form. One copy will be kept by the participants and one returned to the researcher.

The consent form has been designed in accordance with current ethical good practice [28]. The consent form explicitly states that genetic testing is not available as part of this study.

### Withdrawal of subjects

Individuals will be able to withdraw from the process at any time. If individuals wish to withdraw their consent after the interview has taken place, the audio recording and any associated transcript will be destroyed if specifically requested. They will not be contacted for participation in Stage 2 or future research.

### Measures

Semi-structured interview schedules will be developed to address Behavioural, Normative and Control beliefs using the TPB preliminary interview format [26,29], and for probands will also address illness perceptions using an interview schedule based on the IPQ format [22].

For probands, the interview will also address barriers and facilitators to inviting their non-Pagetic relatives to participate in interviews about genetic testing and preventative treatment (a topic guide will be used). For non-Pagetic relatives, the interview will address barriers and facilitators to participating in a programme of genetic testing and preventative treatment (a topic guide will be used).

### Data recording

With the permission of the subject, the entire interview will be digitally audio recorded. The subject will be assigned an anonymous code (study number). Audio recordings will be labelled with the date of interview and study number.

Not all of the interviews will be transcribed. Transcription will occur on a limited selection of interviews (maximum of 5) to allow more detailed analysis. When transcription does take place it will be carried out by a trained data transcriber (not the interviewer). Pauses, including 'hmmms' and 'ahs', and interruptions of one speaker by another will also be indicated in the transcription. Transcribed interviews will be reviewed by the interviewer responsible for data collection (with reference to the original recording) and transcription errors will be rectified. Transcriptions of interviews will follow recognised guidelines and will not include any identifying information.

### Analysis

Data will be content-analysed using simple TPB & IPQ coding frameworks. A sequential approach to sampling will be used whereby data will be analysed throughout recruitment on an ongoing basis until either the point of data saturation is reached (where no new responses emerge) or a maximum of 20 probands and 20 relatives have been interviewed.

### End of study procedures

During the consent process for the study we will ascertain whether participants would like to be sent a report of the study results. For those participants indicating that they would like study results, a short report will be prepared and circulated at the end of the study.

Once the interview is completed the participant will be sent a Thank You letter. This will include a tear-off reply slip that participants will be asked to return indicating whether or not they would like to participate in Stage 2 of the GaP study.

### Confidentiality

Transcripts and audio recordings will be kept in separate locked filing cabinets, and destroyed after 20 years in accordance with current MRC guidelines.

The Study Office, based in the Health Services Research Unit (HSRU), is responsible for the confidentiality of all study records. In accordance with the HSRU code of conduct, all data will be password protected against unauthorised access and stored in accordance with the Data Protection Act 1998. All stored data will be anonymised.

### Finance and indemnity

The study is supported by a grant from the Medical Research Council (MRC). The University of Aberdeen holds the grant, and has accepted Sponsorship responsibilities for the study, including indemnity for negligent and non-negligent harm.

### Reporting and dissemination

A summary of the results of the study will be prepared and distributed, not only to the appropriate funding body, but also to all subjects who take part (if they so wish). Results will be published in peer-reviewed academic journals.

## Abbreviations

**DNA **Deoxyribonucleic acid. The substance that makes up genes.

**Familial Paget's disease **Paget's disease that affects more than one member of a family

**IPQ-R **The Illness Perceptions Questionnaire (R stands for revised version). This measures what people think about an illness in themselves or others.

**MRC **Medical Research Council. A UK government funded organisation that supports medical research [30].

**NARPD **National Association for the Relief of Paget's Disease. A support group for people with Paget's disease, their family and carers [31].

**Non-familial Paget's disease **Paget's disease that only affects one person in a family.

**PDFR **Paget's Disease Family Register. A study led by Prof Ralston, investigating the genetics of Paget's disease.

**PRISM **A trial led by Prof Ralston and co-ordinated by Dr Langston, investigating the treatment of Paget's disease.

**Proband **Initial person of contact within a family who has Paget's disease

**Relative **A relative of a proband (i.e. brother, sister, mother, father, grandmother, grandfather, son, daughter, grandson, grand daughter, nephew, niece)

**SF-36 **A questionnaire that measures general health, and quality of life.

**SQSTM1 **Sequestosome 1 [RNA NM_003900]. One of the genes identified that, when 'faulty', causes Paget's disease.

**SRM **The Self Regulation Model. A psychological theory that aims to identify how people perceive their illness.

**TPB **The Theory of Planned Behaviour. A psychological theory that aims to identify predictors of behaviour using a defined framework.

## Competing interests

Anne Langston received a small travel bursary in 2003 from The Alliance for Better Bone Health, an alliance between Proctor & Gamble, and Sanofi Aventis, which are pharmaceutical companies who manufacture drugs used in the treatment of Paget's disease. She is also a Board Member and Trustee for the National Association for the Relief of Paget's Disease. Stuart Ralston acts as a consultant for Proctor & Gamble, Sanofi Aventis and Novartis, which are pharmaceutical companies who manufacture drugs used in the treatment of Paget's disease. Prof Ralston is also a Board Member and Trustee for the National Association for the Relief of Paget's Disease. Marilyn McCallum is the Chief Executive for the National Association for the Relief of Paget's Disease. There are no competing interests for the other authors.

## Authors' contributions

Anne Langston prepared and wrote the first draft of this protocol. Clare Robertson contributed to the amendments and re-drafting of the protocol to achieve ethical approval. Anne Langston, Marie Johnston, Marion Campbell, Vikki Entwistle, Theresa Marteau, Marilyn McCallum, and Stuart Ralston designed the study and are grant-holders. All authors have read and approved the final manuscript.

## Pre-publication history

The pre-publication history for this paper can be accessed here:



## Figures and Tables

**Figure 2:**
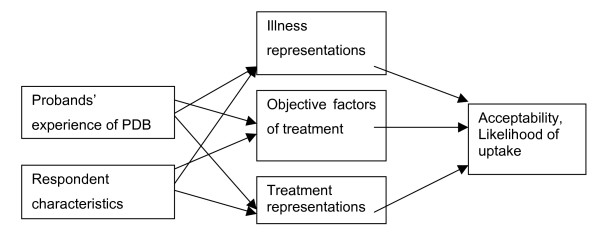
A model of the relationship between explanatory and dependent variables.

**Table 1:** The pool of probands available for the study, and their geographical location.

	Pool of probands available (approx.)
1. Scotland – Aberdeen/Dundee	75
2. Northern England – Manchester/Sheffield/Keighley	160
3. Southern England – London/Southampton	30
